# Could a Haematoma Be Due to an Acquired Phenomenon?

**DOI:** 10.7759/cureus.19792

**Published:** 2021-11-21

**Authors:** Mansoor Zafar, William Butler- Manuel, Joel Newman, Stefano Berliti, Anna Cowley

**Affiliations:** 1 Gastroenterology and Hepatology, Conquest Hospital - East Sussex Healthcare NHS Trust, St. Leonards-on-Sea, GBR; 2 Internal Medicine, Conquest Hospital - East Sussex Healthcare NHS Trust, St. Leonards-on-Sea, GBR; 3 Internal Medicine and Hematology, Conquest Hospital - East Sussex Healthcare NHS Trust, St. Leonards-on-Sea, GBR; 4 Internal Medicine and Acute Medicine, Conquest Hospital - East Sussex Healthcare NHS Trust, St. Leonards-on-Sea, GBR

**Keywords:** prothrombin time (pt), activated partial thromboplastin time (aptt), factor ix, factor viii inhibitor bypassing agents (feiba), coagulation factor viii

## Abstract

Acquired hemophilia, as opposed to congenital hemophilia, develops in individuals with no previous history of bleeding disorder with almost similar numbers of males and females affected. It is predominantly a disease of the elderly. It is an autoimmune disorder and occurs when the immune system produces antibodies that mistakenly attack healthy tissue, specifically the clotting factors, in particular clotting factor VIII. As a result, affected individuals develop abnormal uncontrolled bleeding into the muscles, soft tissues, and the skin and it can occur spontaneously during surgery, or following trauma, and potentially cause life-threatening bleeding complications in severe cases. The affected individuals may remain undiagnosed or be misdiagnosed, making it difficult to determine the actual frequency of the disorder in the general population. The clinical presentation should suspect it with confirmation by an abnormal coagulation test. Typical laboratory results with a recent onset of abnormal bleeding and an isolated prolongation of the activated partial thromboplastin time (APTT), especially in the elderly and peri- and post-partum women, should raise eyebrows. We present two cases following different symptomatology and emphasize the clinical challenges for junior medical doctors who receive patients on the front end. We hope to emphasize understanding simple coagulation blood results followed by a meaningful discussion with the hematology team towards appropriate and timely management of the bleeding diathesis. We hope this case series report will help junior medical doctors manage patients appropriately and consult with their hematology colleagues.

## Introduction

Hemophilia is a predominantly inherited deficiency in coagulation factors causing an increased susceptibility to bleeding. The most commonly affected are coagulation factors VIII and IX, hemophilia A and B, respectively. Hemophilia A and B are inherited in an X-linked fashion where males are affected. However, females are usually asymptomatic carriers but they can get affected if their mother is a carrier and the father is affected. 

Acquired hemophilia is rare, with approximately two new cases per million people in the UK every year and predominantly in middle-aged or elderly patients [1-4]. It affects men and women with no ethnic predilection [5]. Acquired hemophilia results from the spontaneous development of autoantibodies and subsequent deactivation of most commonly factor VIII (FVIII) or occasionally factor IX (FIX) [6,7]. Both factor VIII and IX are essential for thrombin generation through the intrinsic pathway during the coagulation cascade. It can also be associated with solid or hematological cancers, respiratory diseases, ulcerative colitis, dermatological disease, or certain drugs [8]. It can occur in the post-partum period or during the latter stages of pregnancy. In approximately 50% of cases, the cause is idiopathic [1,8].

Patients often present with life-threatening bleeding [8], which is very difficult to control and requires large amounts of replacement coagulation factors. Bleeding into soft tissue can also result in compartment syndrome [9]. Here, we present a case series of two patients with a provisional diagnosis of acquired hemophilia with a history of recent trauma and chronic sepsis but no previous autoimmune disease. Both had resolution of symptoms following management. The formation of antibodies to other coagulation factors is sporadic. The reason for antibody formation against factor VIII is unclear. However, approximately 50% of cases are associated with an underlying disease state, and most cases have a history of an autoimmune phenomenon [10].

## Case presentation

Case 1

A 60-year-old male with an extensive surgical history presented with a five-day history of acute non-traumatic pain, swelling, and bruising in his right upper arm and right calf, giving him difficulties in mobilizing. There were no associated fevers, rigors, mucosal or rectal bleeding, abdominal pain, shortness of breath, or chest pain. He had no previous history of bleeding or thrombosis. He had had a history of sigmoid resection post sigmoid diverticular perforation with a postoperative primary anastomotic leak leading to multiple adhesions and subsequent bowel obstruction. He underwent further resections leading to an end ileostomy, and a few days before admission, he had been experiencing significant purulent discharge from his abdominal wound. His most recent surgical admission was six months before this admission. 

**Figure 1:**
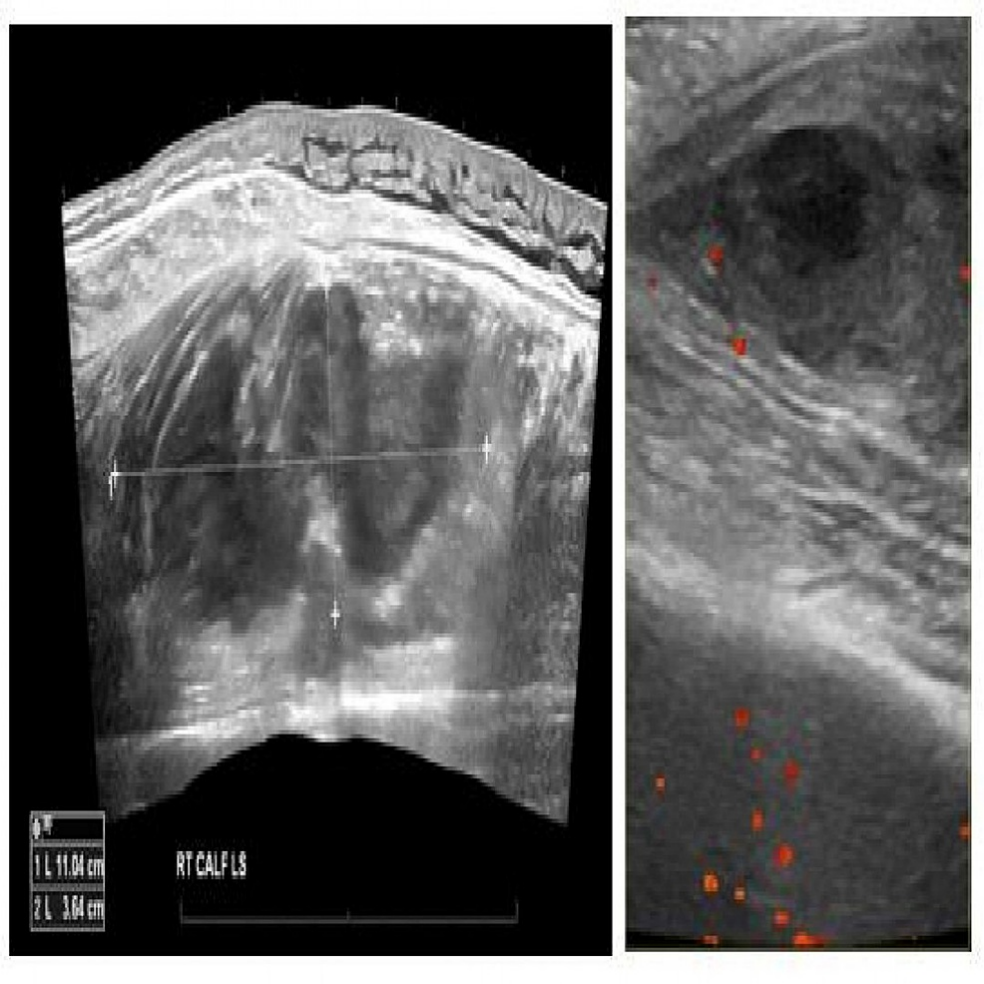
Ultrasonography scan. Left: The right calf demonstrates hematoma, 57 x 45 x 110 mm in length. Right: A large defined intramuscular hypoechoic lesion, 30 x 30 x 85 mm in length, appears to lie within the biceps muscle belly. The appearance is suggestive of hematoma. No thrombus was seen.

Blood tests showed macrocytic anemia, elevated activated partial thromboplastin time ratio (APTT) of 1.87 (normal 0.85-1.10), normal prothrombin time (PT) of 11.2 seconds (10-11.7), and fibrinogen level of 5.1 g/L (1.8-3.6 g/L). Ultrasonography (USG) scanning of his right upper and lower limbs confirmed a large underlying spontaneous hematoma but no thrombus (Figure 1).

He was reviewed by the hematology team whose impression following the mixing studies was of acquired hemophilia. Further samples were sent for FVIII and FIX levels, and an inhibitor screen (Table 1). He was transferred to the hematology ward where he was treated with fresh frozen plasma, red blood cells, vitamin K and tranexamic acid. Once the low FVIII level was confirmed, he received daily factor eight inhibitor bypassing agent (FEIBA) infusions.

**Table 1:** Serial blood tests with coagulation profiles with improvement in factor VIII inhibitor levels while on treatment. Source: Haematology Pathology Laboratory, East Sussex Healthcare NHS Trust * Incomplete correction to 1.24 in 50:50 mix with normal plasma. 'INR therapeutic range for warfarin: 2.0 - 4.5 “APTR therapeutic range for heparin: 1.5 - 2.5 ! Test performed at St Thomas' Hospital (Viapath)

	Units	Range	Day 1	Day 2	Day 3	Day 8	Day 13	Day 21	Day 41	Day 57
Hemoglobin	g/L	130- 180	82	77	87	-	-	99	122	124
Mean cell volume	fl	80-100	106.5	104.9	106.2	-	-	96.7	91.6	92.1
White cell count	x 10^9^/L	4-11	9.08	6.5	6.57	-	-	6.61	6.85	6.19
Neutrophils	x 10^9^/L	2-7.5	5.60	3.92	4.16	-	-	4.67	3.69	2.68
Platelet count	x 10^9^/L	150-400	267	261	265	-	-	230	333	206
Prothrombin time (PT)	seconds	10-11.7	11.2	9.5	9.4	-	-	-	10.9	10.4
^'^International normalization ratio (INR)	-	0.8-1.2	1.1	0.9	0.9	-	-	-	1.0	1.0
^''^Activated partial thromboplastin ratio (APTR)	-	0.85-1.10	1.87^*^	1.56	1.58	-	-	1.92	1.82	1.57
Fibrinogen level	g/L	1.8-3.6	5.1	4.8	4.8	-	-	-	-	-
Factor VIII assay	iu/dl	50.0-150.0	7	7.6	7.8	5.4	4.2	5.8	6.8	13.4
Factor IX assay	Iu/dl	50.0-150.0	132.2	-	-	-	-	-	-	-
^!^Factor VIII inhibitor (Human)	NBU/ml	-	-	-	-	-	19.5^!^	27.84^!^	20.64	7.20

Due to the risk of compartment syndrome, he was reviewed by the orthopedic team who advised observation and conservative management with mobilization and analgesia. The swelling gradually reduced throughout admission following ongoing daily FEIBA replacement. His abdominal wound discharge was investigated with CT scanning, which revealed an organizing collection/abscess within the left anterior abdominal wall indistinguishable from a large bowel loop, raising concern for fistulation. The surgical team reviewed it; they had no concerns requiring acute intervention but advised further evaluation with MRI-enterography (MRE). This identified no definite fistula but confirmed an abdominal wall/subcostal fluid collection that was subsequently aspirated by interventional radiology under ultrasound guidance. He completed a course of intravenous (IV) antibiotic therapy to cover for associated infection. Following drainage of his intra-abdominal collection, his case was re-reviewed by hematology in the multi-disciplinary meeting (MDM), and he was deemed appropriate for weekly rituximab therapy for four weeks. Steroids were avoided initially due to intra-abdominal sepsis. He was eventually found to be medically fit for discharge with follow-up in the daycare unit to monitor FVIII and inhibitor levels and FEIBA replacement. After discussion with a tertiary center, he was also prescribed a modest weaning dose of steroids with proton pump inhibitors (PPI).

Case 2

**Figure 2:**
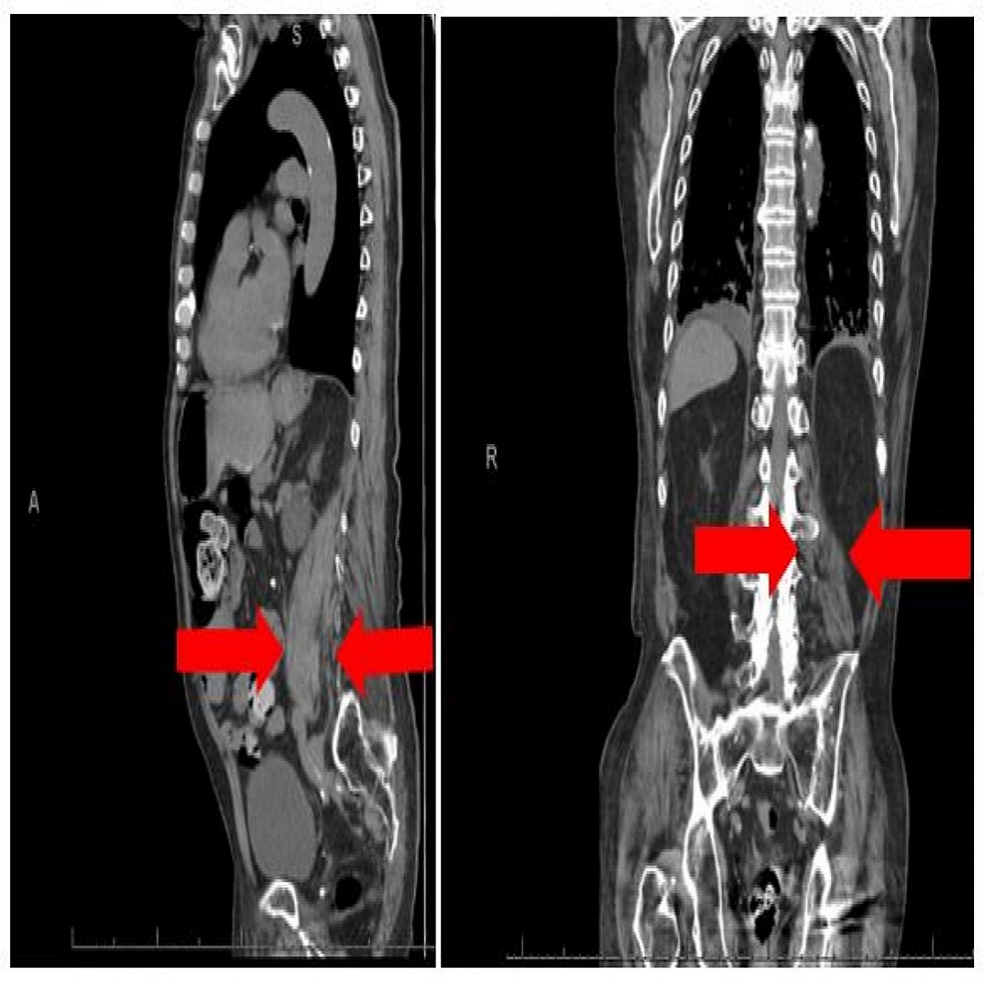
Computed tomogram (CT) with contrast; sagittal and coronal views. Red arrows show large extended left iliopsoas hematoma.

An 88-year-old male with a history of atrial fibrillation (AF) on apixaban, hypertension, bilateral cataracts, chronic kidney disease (CKD), and a left hip hemiarthroplasty was admitted under the orthopedic team complaining of progressively worsening back pain following a fall. A urinary bladder scan suggested he had urinary retention of around 750 ml, so he was catheterized. This revealed haematuria, and initially, it was thought to be due to apixaban, which was withheld. The CT-abdomen-pelvis demonstrated an extended left iliopsoas hematoma (Figure 2).

His blood test results also showed an acute kidney injury (AKI) which responded to intravenous fluid resuscitation. Coagulation studies were performed, which revealed an activated partial thromboplastin ratio (APTR) of 3.90 (0.85-1.10) and a PT of 11.5 seconds (10-11.7). His case was discussed with the hematology team who concluded following the mixing studies that it was acquired hemophilia due to his pattern of bleeding with elevated APTT and not due to apixaban use (Table 2).

**Table 2:** Serial blood tests with coagulation profiles with improvement in VIII inhibitor levels while on treatment. Source: Haematology Pathology Laboratory, East Sussex Healthcare NHS Trust * Incomplete correction to 1.24 in 50:50 mix with normal plasma. 'INR therapeutic range for warfarin: 2.0 - 4.5 “APTR therapeutic range for heparin: 1.5 - 2.5 ! Test performed at St Thomas' Hospital (Viapath)

	Units	Range	Day 1	Day 2	Day 5	Day 8	Day 13	Day 19	Day 40	Day 49
Hemoglobin	g/L	130-180	102	103	113	114	113	118	117	-
Mean cell volume	fl	80-100	92.4	94.7	93.2	94.1	94.3	93.7	92.7	-
White cell count	x 10^9^/L	4-11	17.65	13.42	15.53	16.53	13.97	13.65	11.54	-
Neutrophils	x 10^9^/L	2-7.5	16.05	10.58	10.95	12.98	10.87	11.15	8.97	-
Platelet count	x 10^9^/L	150-400	522	503	336	197	128	121	149	-
Prothrombin time (PT)	Seconds	10-11.7	11.5	11.8	12.4	12.4	12.0	11.5	11.4	-
^'^International normalization ratio (INR)	-	0.8-1.2	1.2	1.1	1.1	1.1	1.1	1.1	1.1	-
^''^Activated partial thromboplastin ratio (APTR)	-	0.85-1.10	3.90^*^	2.26	1.33	1.18	1.09	1.11	1.07	-
Fibrinogen level	g/L	1.8-3.6	5.0		-	-	-	-	-	-
Factor VIII assay	iu/dl	50.0-150.0	<0.1	7.3	25.4	40.0	31.2	42.9	47.0	53.7
Factor IX assay	iu/dl	50.0-150.0	107.4	-		-	-	-	-	-
Factor XI assay	iu/dl	57.9-118.5	59.3	-	-	-	-	-	-	-
^!^Factor VIII inhibitor (Human)	NBU/ml	-	32.6	9.20	3.3	1.0	1.7	1.8	0.83	<0.70

Further blood tests confirmed a very low FVIII and high inhibitor level. He received six units of red blood cells over the following 11 days and FEIBA while apixaban was put on hold. He also received prednisolone 1mg/kg and cyclophosphamide 50mg od. A repeat CT was done which showed a slight improvement in the iliopsoas hematoma. His factor VIII level eventually normalized with steroids and cyclophosphamide, and he was eventually discharged home.

## Discussion

In patients with acquired hemophilia, the history is usually a sudden onset of bleeding in patients with no previous coagulation problems. The bleeding patterns are different from congenital hemophilia with spontaneous hemarthrosis being particularly rare [9], including purpura and bleeding into muscle and mucosal membrane hemorrhages. Also common are intracerebral and gastrointestinal bleeding, haematuria, intra-abdominal bleeding, postpartum hemorrhages, and postoperative bleeding [9]. The condition is often not recognized or is mistaken for other coagulation disorders such as disseminated intravascular coagulation [9]. It is differentiated by an elevated APTT, PT and fibrinogen, and normal platelet count.

A meta-analysis of 249 cases after a median follow-up of 12 months showed a complete remission rate of 74% [11]. In the same study, poor prognostic factors were age >65 years, no remission, and the nature of the underlying disease, malignancy being the worst. Also, a common association is soft tissue bleeding [12]. The mortality rate in acquired hemophilia ranges from 8% to 22%. Bleeding is the most common cause of mortality in patients with acquired hemophilia, and hemorrhage is more severe than in congenital hemophilia. The highest mortality risk is within the first few weeks of presentation [13]. 

The standard approved treatment regimen for acquired hemophilia is two-fold. The first is to administer hemostatic therapy. The second is to administer immunosuppression with steroids and cyclophosphamide to eliminate the autoantibodies inhibiting factor VIII in a high percentage of pregnancy or drug-related acquired hemophilia. The antibodies disappear spontaneously after an average period of 30 months [14].

There are 13 factors in the coagulation process, and coagulation occurs when the clotting factors are activated in the correct order. The coagulation process can be prolonged or prevented if any of the factors are missing or blocked. For a stable clot to form, the deficient factor in the coagulation process should be bypassed or replaced. This is the principle underlying the recommended hemostatic therapies. The first-line treatment for bleeding in acquired hemophilia should be either activated prothrombin complex concentrate (APCC), FEIBA, or recombinant activated factor VII (rFVIIa, 'Novoseven') [14]. Factor eight inhibitor bypassing activity (FEIBA) is a mixture of activated coagulation factors (II and Xa) that convert prothrombin to thrombin in the coagulation pathway without needing FVIII or FIX, the factors affected in hemophilia A and B. Factor eight inhibitor bypassing activity (FEIBA) can thus bypass the effects of the inhibitory antibodies, normalizing the coagulation process. Recombinant factor VIIa 'Novoseven' is also a bypassing agent which activates factor X directly, skipping the need for factor VIII or IX.

Both treatments reported side effects, including venous thrombotic events, sepsis, and myocardial infarction [14]. If the initial choice (usually FEIBA) fails, the alternative ('Novoseven') should be given. There should be no delay in starting immune therapy to eradicate autoantibodies, and the combination of cyclophosphamide and oral steroids is the treatment of choice. Rituximab is suggested as a promising drug for treating acquired hemophilia but is currently recommended as a second-line treatment option [14]. However, it can be used first-line if there are contraindications to immunosuppressive treatment, as seen in the first case.

Lastly, for the front-line physicians who come across such scenarios, here's a brief outline of the approach to be adopted: Once the prolonged APTT is the only abnormality found as a general rule, a mixing study is the next step. The serum with a normal APTT must be mixed in a similar proportion (50:50) with the serum with the abnormal APTT. The resulting APTT may be normalized, implying that the normal APTT serum has replaced a factor missing during the mixing study. If it is not normalized (it is a time-dependent process, and therefore can appear normal initially but then will become prolonged), it implies the presence of an antibody destroying the factor being added in the mixing process. Therefore, if the APTT is still prolonged after the mixing study, there are two possibilities - firstly, either there's a presence of factor VIII Inhibitor or, secondly, there's a presence of antiphospholipid antibodies. Although technically further studies could differentiate the two, clinical history is often enough. People with factor VIII inhibitors present with a history of bleeding (such as after the dental extraction in our patient), whereas the antiphospholipid syndrome has a history of clots/thrombosis, although both present with the same abnormality, APTT prolongation [15,16].

## Conclusions

Although this is very familiar to the hematology teams, for those involved in acute medical care, the presentation of acquired hemophilia is an important consideration for a patient presenting with bleeding and an isolated prolonged APTT. Mixing studies are helpful to demonstrate that the prolonged APTT does not correct with normal plasma. Early liaison with hematology is essential for patients to receive hemostatic therapy to stop the bleeding and immunosuppressive treatment to eradicate the inhibitor.
